# Deep Learning Algorithms for the Prediction of Posttransplant Renal Function in Deceased-Donor Kidney Recipients: A Preliminary Study Based on Pretransplant Biopsy

**DOI:** 10.3389/fmed.2021.676461

**Published:** 2022-01-18

**Authors:** You Luo, Jing Liang, Xiao Hu, Zuofu Tang, Jinhua Zhang, Lanqing Han, Zhanwen Dong, Weiming Deng, Bin Miao, Yong Ren, Ning Na

**Affiliations:** ^1^Department of Kidney Transplantation, The Third Affiliated Hospital of Sun Yat-sen University, Guangzhou, China; ^2^Department of Pathology, The Third Affiliated Hospital of Sun Yat-sen University, Guangzhou, China; ^3^Artificial Intelligence Innovation Center, Research Institute of Tsinghua, Pearl River Delta, Guangzhou, China; ^4^Guangdong Provincial Key Laboratory of Digestive Cancer Research, The Seventh Affiliated Hospital of Sun Yat-sen University, Shenzhen, China

**Keywords:** kidney transplantation, deceased donor, graft function, deep learning, whole slide digital image

## Abstract

**Background:**

Posttransplant renal function is critically important for kidney transplant recipients. Accurate prediction of graft function would greatly help in deciding acceptance or discard of allocated kidneys.

**Methods:**

**:** Whole-slide images (WSIs) of H&E-stained donor kidney biopsies at × 200 magnification between January 2015 and December 2019 were collected. The clinical characteristics of each donor and corresponding recipient were retrieved. Graft function was indexed with a stable estimated glomerular filtration rate (eGFR) and reduced graft function (RGF). We used convolutional neural network (CNN)-based models, such as EfficientNet-B5, Inception-V3, and VGG19 for the prediction of these two outcomes.

**Results:**

In total, 219 recipients with H&E-stained slides of the donor kidneys were included for analysis [biopsies from standard criteria donor (SCD)/expanded criteria donor (ECD) was 191/28]. The results showed distinct improvements in the prediction performance of the deep learning algorithm plus the clinical characteristics model. The EfficientNet-B5 plus clinical data model showed the lowest mean absolute error (MAE) and root mean square error (RMSE). Compared with the clinical data model, the area under the receiver operating characteristic (ROC) curve (AUC) of the clinical data plus image model for eGFR classification increased from 0.69 to 0.83. In addition, the predictive performance for RGF increased from 0.66 to 0.80. Gradient-weighted class activation mappings (Grad-CAMs) showed that the models localized the areas of the tubules and interstitium near the glomeruli, which were discriminative features for RGF.

**Conclusion:**

Our results preliminarily show that deep learning for formalin-fixed paraffin-embedded H&E-stained WSIs improves graft function prediction accuracy for deceased-donor kidney transplant recipients.

## Introduction

Kidney transplantation remains the best option for patients with end-stage renal disease (ESRD). However, due to a shortage of organs, the use of marginal kidneys is rising (1), and as a result, the incidence of complications is gradually increasing. Graft function (estimated glomerular filtration rate [eGFR]) is one of the most crucial risk factors for surgical complications and long-term survival. It has been indicated that kidney function after transplantation correlates strongly with graft long-term survival (2–4). Graft function is the core cornerstone linked to long-term survival and plays a crucial role in assessing transplant results (5, 6). Posttransplantation graft function is determined by the donor, procurement and preservation, recipient characteristics, and postsurgical treatment, though there is currently no stable model for predicting allograft functional outcomes. Pretransplant graft discard is influenced by the experience of transplant surgeons based on donor characteristics, with or without biopsy evaluation, and assessment of the donor kidneys mainly involves clinical scores and histological evaluation. In general, clinical score evaluation began with standard criteria donor (SCD)/expanded criteria donor (ECD) binary classification and has evolved to the currently widely used kidney donor risk index (KDRI). The discriminative power of such a clinical score is low to moderate, and the use of KDRI in the United States leads to a high discard rate of donated kidneys. Indeed, in our clinical practice, low-KDRI kidneys do not always regenerate satisfactory graft function, whereas many high-KDRI kidneys regenerate very good renal function. Overall, the predictive performance of using net clinical features for graft function outcome is relatively poor (7), though the addition of histopathological features may improve the predictive performance of graft outcome. Pretransplant histological evaluation of the donor kidneys is critically important for kidney quality evaluation, especially for marginal kidneys. The histological score composites include the glomerulus, tubule, interstitium, and vessels (mainly artery). Nonetheless, the usefulness of such a score remains controversial due to its low predictive power for transplant outcomes (8, 9).

Pretransplant biopsy by H&E staining is crudely evaluated for acute kidney injury and glomerular sclerosis, depending on the experience of the pathologist, and is variable. Pathological evaluations of the donor kidneys from different pathologists/nephrologists and from different levels of pathologists disagree. For example, a previous study showed good reproducibility regarding glomerulus number and sclerosis percentage but very poor or fair intraclass correlation regarding interstitial fibrosis, tubular atrophy, interstitial inflammation, arteriolar thrombi, and arterial intimal fibrosis, especially for slides from needle and frozen biopsies. The Banff Working Group suggests that training of general pathologists to assess donor biopsies using consistent criteria should be pursued, and adoption of rapid formalin-fixation and paraffin-embedding protocols may potentially reduce interference for frozen slides (10). These recommendations might increase the reliability of histopathological evaluation, and an automatic analytic machine with deep learning algorithms may assume this role in analyzing the donor biopsies.

The use of artificial intelligence (AI) is increasing explosively in the medical field. Deep learning methods, such as convolutional neural networks (CNNs), are very practical for image and audio analysis (11). Inspired by these ideas, we aimed to explore whether the combination of kidney biopsy whole-slide images (WSIs) and clinical features can provide more information for transplant outcome prediction.

## Materials and Methods

### Study Population and Clinical Variable Data

**Figure 1:**
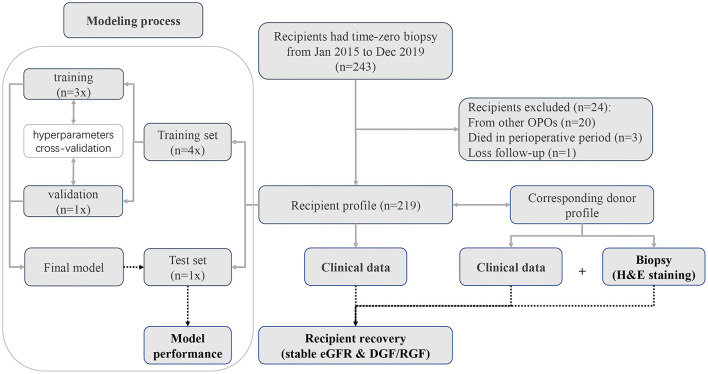
Patient recruitment diagram. In total, pretransplantation biopsy slides were available for 243 recipients. Twenty recipients were excluded because the donors were from other organ procurement organizations (OPOs). Three recipients died during the perioperative period. One recipient was lost to follow-up. Finally, we included 219 recipients with complete data for deep learning analysis.

Deceased-donor kidney recipients at the Third Affiliated Hospital of Sun Yat-sen University, Guangzhou, China from January 2015 to December 2019 were retrospectively reviewed. In total, 243 donor kidney recipients underwent pretransplantation biopsy. Demographic data for the recipients and donors were collected from China Organ Transplant Response System (COTRS) and the hospital information system (HIS). COTRS is the sole legitimate official registry platform designated by the National Health Commission of China for solid organ donation from deceased-citizen donors, matching, and allocation. After screening, 219 recipients were included in the final analysis. The screening process is depicted in Figure 1. This study was approved by the research ethics committee (IRB No. [2020]02-243-01) and was compliant with the Declaration of Helsinki. The clinical and research activities being reported are consistent with the Principles of the Declaration of Istanbul as outlined in the “Declaration of Istanbul on Organ Trafficking and Transplant Tourism.”

Delayed graft function (DGF) was defined as requiring dialysis within the first week after transplantation (12). Slow graft function (SGF) was defined as serum creatinine at postoperative day 7 (POD7) ≥ 2.5 mg/dl, and immediate graft function (IGF) was defined as creatinine at POD7 <2.5 mg/dl (13). Reduced graft function (RGF) was a composite endpoint consisting of DGF and SGF. The renal function of each recipient was indexed with the eGFR and comprehensively evaluated based on serum creatinine (or calculated eGFR) based on each follow-up monitoring visit within the first year. In this study, we used stable eGFR as the graft outcome. The eGFR of recipient was calculated by the Chronic Kidney Disease-Epidemiology (CKD-EPI) algorithm (14). Stable eGFR was defined as the approximate median value where the monitoring results fluctuated within the first year (3~12 months). Stable eGFR was evaluated from the best period that the graft function of recipient was reached by two senior physicians and not the whole year of monitoring results. Recipients who died during the perioperative period, accepted donations from other organ procurement organizations (OPOs), or were lost to follow-up were excluded. The case of a recipient with a primary non-functional kidney was labeled with the last eGFR before returning to dialysis. Additionally, we arbitrarily divided stable graft function into binary categories by a cutoff of 45 ml/min/1.73 m^2^ as sensitivity analysis. Based on previous publications, the graft failure hazard rises sharply over this point (2–4, 7, 15). The clinical characteristics screened for the predictive model building included both donor and recipient features. These features included recipient age, sex, height, weight, transplant history, primary cause of ESRD, diabetes, dialysis modality, dialysis vintage, positive panel reaction antibody (PRA), human leukocyte antigen (HLA) mismatch level, and donor characteristics of cold ischemia hours, warm ischemia minutes, cause of death, death type, donated kidney, donor sex, age, height, weight, terminal serum creatinine level, and KDRI. Missing values in the HLA mismatch level were deemed one category.

### Biopsy Samples and Model Training

#### Pathological WSIs

We reviewed all H&E-stained (formalin-fixed paraffin-embedded: FFPE) pretransplantation biopsy slides and scanned and stored the WSIs at × 200 magnification with a resolution of 0.23 μm/pixel using an automatic digital slide scanner (Panoramic 250 FLASH, 3DHISTECH Ltd, Budapest, Hungary). In total, 219 recipients with H&E-stained slides of the donor kidneys were included in the final analysis. All biopsies were procedurally performed using the 18-gauge needles during donor kidney trimming (i.e., all samples were preimplantation biopsies). Pathological scores (Remuzzi scores) were evaluated by a single pathologist (Jing Liang).

#### Deep Learning and Transfer Learning

**Figure 2:**
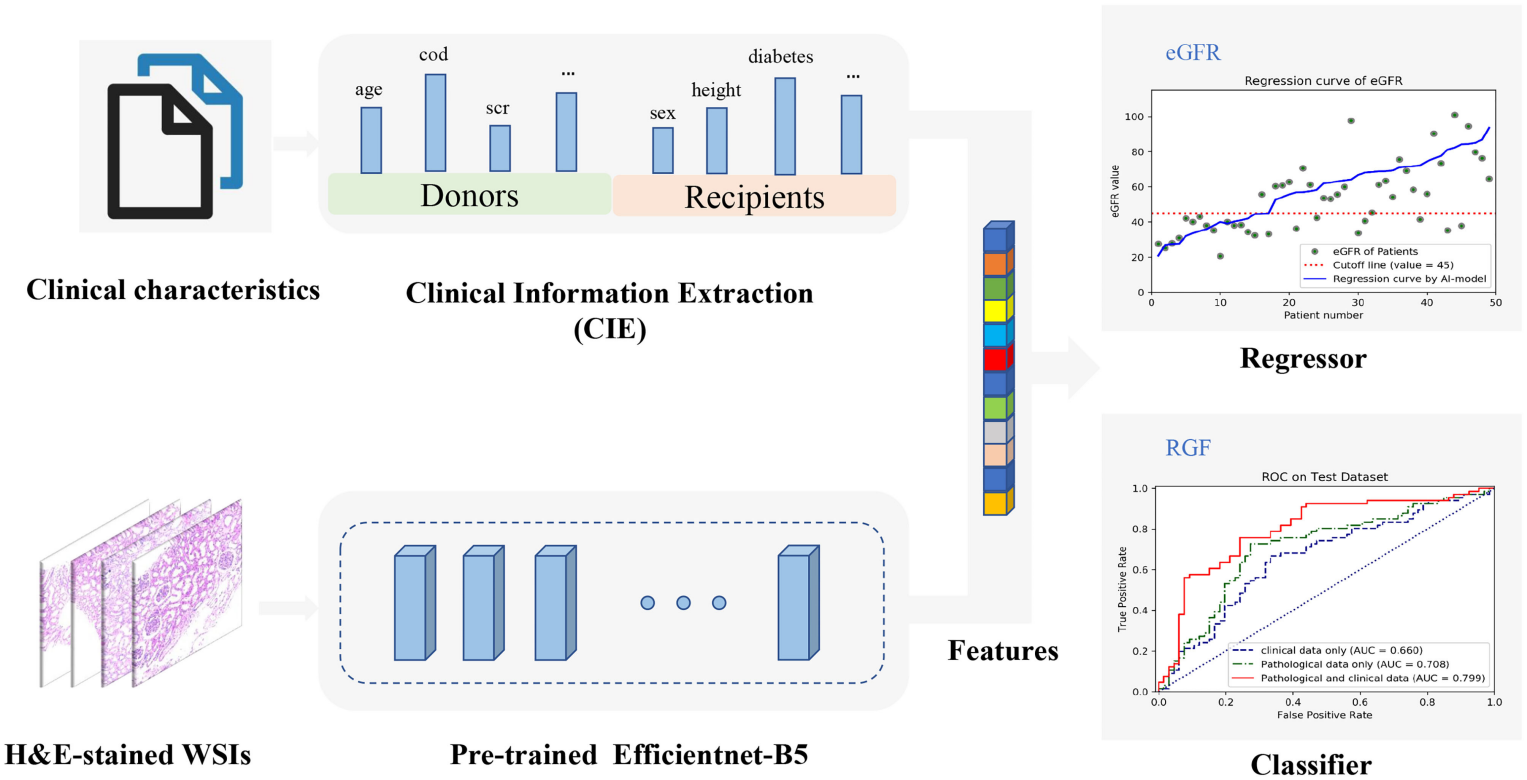
The overall workflow diagram of the model. H&E-stained whole-slide images (WSIs) are fed into the parallel pretrained Efficientnet-B5 model to extract features automatically which can be combined with all clinical information encoded from clinical texts. Then, the combined features can be used to construct a regression model to predict estimated glomerular filtration rate (eGFR) values and a classifier to predict reduced graft function (RGF), simultaneously.

A CNN-based model was used to extract the features of the H&E WSIs, such that important information could be obtained for regression fitting. EfficientNets is a new baseline network designed by neural architecture search and consists of a family of models. From B0 to B7, the corresponding accuracy increases, but the number of parameters also increases, which leads to a decrease in training and deployment efficiency. The EfficientNet B5 model was considered to be the most suitable base model due to the trade-off between few parameters and sufficiently high ImageNet Top-1 accuracy (Top-1 accuracy means that the model with the highest probability of prediction must be exactly the true classification). Correspondingly, Top-5 accuracy indicates that any part of the model that yields the 5 highest probability predictions should include the true classification. To find the most suitable model for predicting stable eGFR and RGF, the performances of EfficientNet-B5, Inception-V3 and VGG19 were compared, and EfficientNet-B5 was selected as the base network due to its superior performance. Regions of interests (ROIs) patches selected from H&E-stained renal biopsy tissues were first sent into EfficientNet-B5 to extract high-dimensional pathological image features, and the corresponding clinical texts of patient preprocessed by one-hot coding conversion and normalization were then synchronously input into a fully connected network (FCN) to extract the clinical information. The whole process is illustrated in Figure 2.

To perform feature fusion, the extracted clinical information (CIE), i.e., clinical data, was spliced with the penultimate layer of the EfficientNet-B5 network; next, a new layer containing only one neuron was added to regress the eGFR values, and a parallel layer containing two neurons was added to classify the RGF state. It should be noted that for regression tasks, the neurons in the last layer do not use any activation function such that the model can output unlimited prediction values. This model, which includes clinical information and a CNN, is called the EfficientNet-B5 + CIE model, where the CIE model denotes the clinical information extraction model. After obtaining the regression model, we classified the actual value and predicted value according to cutoff values (eGFR of 45 ml/min/1.73 m^2^). With the help of the RGF classification model and the indirect eGFR classification model, postoperative recovery can be qualitatively evaluated. In addition, the sample size of medical pathological images is relatively small compared with the ImageNet dataset (http://www.image-net.org/). To avoid overfitting, we used transfer learning to initialize all parameters of the CNN model with ImageNet trained parameters before training and then updated the parameters *via* backpropagation during training. In conclusion, we used both clinical data and histopathological images to build neural network models to predict stable eGFR and RGF. We preset to test six prediction models, such as a model based on clinical data (CIE model), a model based on clinical data and the histopathological Remuzzi score (CIE+PRS model), models based on histopathological images (VGG19 model, Inception-V3 model, and EfficientNet-B5 model), and a model based on clinical data and histopathological images (EfficientNet-B5+CIE model).

#### Implementation Details

**Figure 3:**
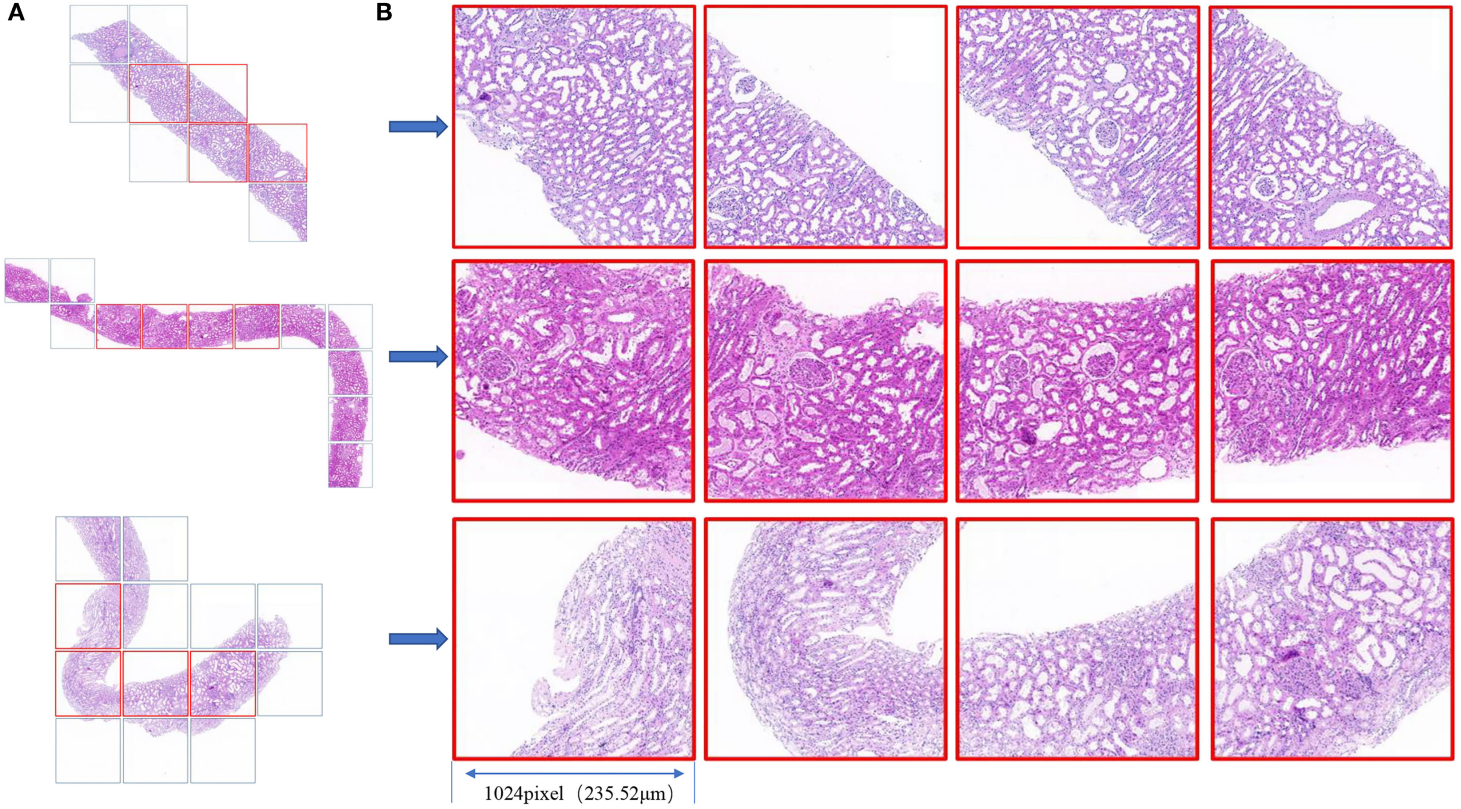
**(A)** Biopsy tissue was scanned as a WSI under a × 20 objective lens, and the slide window with a 1,024 window width was used for tiling the WSI to patches (square box). **(B)** The four patches with the largest proportion of pathological tissue in each WSI are framed in red. The field of view of each square patch is 1,024 × 1,024 pixels, corresponding to 235.52 × 235.52 μm^2^.

We used Python 3.6 as the programming language and Keras 2.2 as the deep learning architecture. We trained and tested our models using one Nvidia Tesla V100 GPU with 32 GB memory with the help of NumPy, Matplotlib, and scikit-learn. Cases with each H&E-stained WSI were randomly divided into a training dataset, validation dataset, and independent test dataset at a ratio of 3:1:1. First, we used the validation dataset to choose hyperparameters, such as the number of epochs and the learning rate. After the parameters were determined, the validation data and training data were combined to form a new training dataset for retraining the model, and the performance of the model was evaluated using the independent test dataset (as shown in Figure 1). Four patches with the largest proportion of pathological tissue in each H&E-stained WSI were selected as ROIs through OpenSlide 1.9 using a sliding window with window width set as 1,024 pixels (as shown in Figure 3). A total of 876 patches were obtained. Before inputting the models, the resolution was adjusted to 256 pixels and normalized. Our models adopted the Adam optimizer with a learning rate of 8e-4. The best model was saved when the mean absolute error (MAE) for the test dataset was the lowest in 500 epochs.

#### Model Performance Evaluation Metrics

For regression prediction tasks, the MAE, root mean square error (RMSE), R-squared value, and explained variance score (EVS) were calculated. The MAE is the mean value of the absolute value of error between the real value and the predicted value. The RMSE is the square of the difference between the real value and the predicted value; the sum is averaged, and the square root is obtained. The R-squared value and EVS represent the degree of fitting, and the values range from 0 to 1. The higher the value is, the better the fit is.

For classification prediction tasks, the confusion matrix and receiver operating characteristic (ROC) curve were generated. The confusion matrix is used to visually evaluate the performance of deep learning algorithms. The sensitivity, specificity, positive predictive value, and negative predictive value can be calculated by means of the confusion matrix. The ROC curve is depicted by plotting the true positive rate (TPR, sensitivity) vs. the false-positive rate (FPR, 1-specificity) at various threshold settings. Accuracy is assessed by the area under the ROC curve (AUC).

### Statistical Analysis

The demographics of the donors and recipients are presented as frequencies for categorical variables and medians (interquartile ranges [IQRs]) for continuous variables. Differences were explored using the Wilcoxon rank-sum tests (skewed distribution) or the *t*-test (normal distribution) for continuous variables and Fisher's exact tests for categorical variables. The statistical analyses were performed using R 4.0.2 (R Foundation for Statistical Computing, Vienna, Austria).

## Results

### Population Characteristics

**Figure 4:**
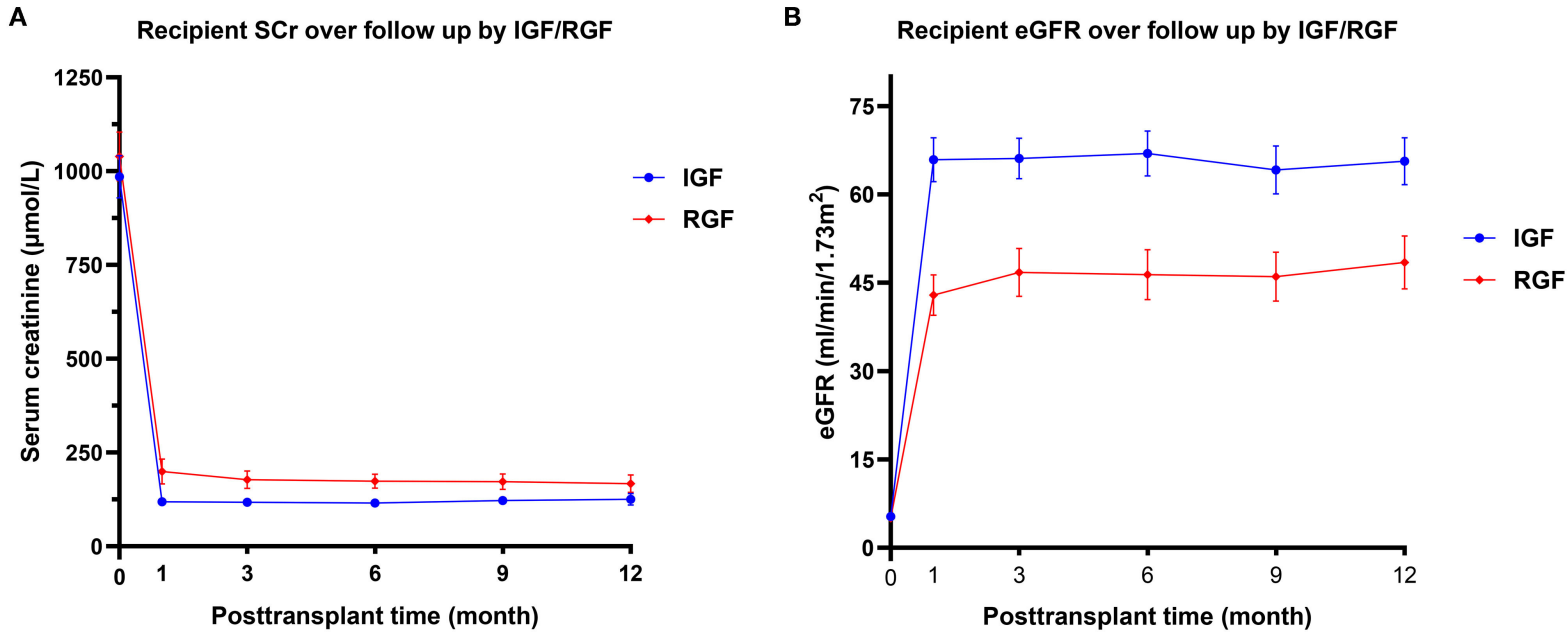
Recipient serum creatinine **(A)** and eGFR **(B)** over the first year follow up by immediate graft function (IGF)/RGF. The values of *p* between IGF and RGF groups at each time point were adjusted by the Holm-Sidak method. Differences in pretransplant serum creatinine and eGFR are not significant, but are significantly different at each time point (adjusted *p* < 0.01).

In total, 219 recipients underwent donor kidney biopsy, and complete follow-up was included in the analysis. All recipients were prescribed traditional triple immunosuppression regimens (CNI+ MPA+ glucocorticoids). The features used for the predictive model building are shown in Table 1. In this population, the incidence of DGF and RGF was 25/219 (11.4%) and 92/219 (42%), respectively. Due to the large imbalance in DGF proportion, we did not set DGF as an analytical target for deep learning and used RGF instead. The median stable eGFR was 61.2 [47.1, 76.9] ml/min/1.73 m^2^ during follow-up visits. Donor death category, donor cause of death KDRI, terminal creatinine, warm ischemia minutes, cold ischemia hours, recipient sex, weight, Remuzzi scores, and stable eGFR were significantly different between the RGF groups. Compared with RGF recipients, the IGF group had better stable eGFR, lower body weight, a higher proportion of female recipients, lower warm ischemia minutes and cold ischemia hours, lower donor KDRI and terminal creatinine levels, and a higher proportion of trauma donors and DBD donors. These results were consistent with previous results. Figure 4 displays the serum creatinine level (Figure 4A) and eGFR level (Figure 4B) of two recipient groups at each time point during the first year follow-up. The trend curve shows better graft function recovery in IGF recipients than in RGF recipients.

**Table 1:** Distributions of the patient baseline characteristics.

**Characters**	**IGF (*N =* 127)**	**RGF (*N =* 92)**	**Total (*N =* 219)**	***P*-value**
**Stable eGFR**	68.8 (55.4, 82.4)	47.4 (38.0, 62.1)	61.2 (47.1, 76.9)	**<** **0.01**
**DGF**				**<** **0.01**
Yes	0	25	25	
No	127	67	194	
**Donor**				
Age	44 (34, 52)	44 (37, 51)	44 (35, 51.5)	0.44
Sex				0.62
Female	25	21	46	
Male	102	71	173	
Weight (kg)	60 (55, 70)	65 (55, 75)	64 (55, 71)	0.31
Height (cm)	167 (162, 172)	168 (160, 170)	168 (161, 172)	0.75
Donor death				**0.006**
DCD	73	70	143	
DBD	54	22	76	
Donor type				0.683
SCD	112	79	191	
ECD	15	13	28	
Cause of death				**<** **0.01**
CVD	50	59	109	
Trauma	70	27	97	
Other	7	6	13	
KDRI	1.31 (1.09, 1.53)	1.50 (1.34, 1.71)	1.40 (1.21, 1.62)	**<** **0.01**
Terminal creatinine (μmol/L)	103 (70, 165)	187 (103, 301)	120 (76.5, 200)	**<** **0.01**
Kidney				0.49
Left	61	49	110	
Right	66	43	109	
**Match and preservation**				
HLA mismatch level				0.31
Level 1	0	0	0	
Level 2	2	1	3	
Level 3	6	10	16	
Level 4	86	62	148	
Missing	33	19	52	
PRA				0.53
Positive	13	12	25	
Negative	114	80	194	
CIT (h)	7.7 (6.7, 9.5)	8.9 (7.3, 10.9)	8.2 (6.9, 10.2)	**0.01**
WIT (min)	6 (0, 10)	10 (0, 11)	7 (0, 10)	**0.02**
**Recipient**				
Age	40 (34, 49)	41.5 (33, 48)	40 (33.5, 48.5)	0.88
Sex				**0.01**
Female	45	17	62	
Male	82	75	157	
Weight (kg)	58 (52, 67.3)	60 (54.5, 70.3)	60 (53.5, 69.8)	**0.05**
Height (cm)	168 (162, 170)	168 (165, 171)	168 (163, 170)	0.07
Transplantation history				0.24
Yes	2	4	6	
No	125	88	213	
Cause of ESRD				0.32
Glomerulonephritis	107	74	181	
DN	6	10	16	
HTN	5	4	9	
Others	9	4	13	
Diabetes				0.68
Yes	15	13	28	
No	112	79	191	
Dialysis modality				0.11
HD	83	71	154	
PD	28	16	44	
No dialysis	16	5	21	
Dialysis vintage				0.33
No dialysis	16	5	21	
1~6 months	34	24	58	
7~12 months	28	22	50	
> 12 months	49	41	90	
CNI				**0.03**
Tac	110	69	179	
CsA	17	23	40	
MPA	127	92	219	1.00
Glucocorticoids	127	92	219	1.00
**Pathology (Remuzzi score)**				
*Glomerular global sclerosis*				**<0.01**
0	67	41	108	
1	50	27	77	
2	10	20	30	
3	0	4	4	
*Tubular atrophy*				**<0.01**
0	79	34	113	
1	48	58	106	
2	0	0	0	
3	0	0	0	
*Interstitial fibrosis*				0.06
0	103	64	167	
1	24	27	51	
2	0	1	1	
3	0	0	0	
*Arterial and arteriolar narrowing*				**0.01**
0	96	52	148	
1	27	29	56	
2	4	10	14	
3	0	1	1	

*DGF, delayed graft function; DBD, donation after brain death; DCD, donation after circulatory death; CVD, cerebrovascular disease; CNS, central nervous system; KDRI, Kidney Donor Risk Index (donor-only); HLA, human leukocyte antigen; PRA, panel reaction antibody; CIT, cold ischemia time; WIT, warm ischemia time; ESRD, end-stage renal disease; DN, diabetic nephropathy; HTN, hypertensive nephropathy; HD, hemodialysis; PD, peritoneal dialysis. Tac, tacrolimus; CsA, cyclosporine A; MPA, mycophenolic acid. Missing HLA mismatch levels usually occurred at emergent procurement, and donor HLA results were not registered in COTRS*.

*Differences between groups were evaluated using Mann–Whitney U-tests for continuous variables and Fisher's exact tests for categorical variables. Bold values indicate the statistical significance*.

### Prediction of Graft Function Recovery

**Figure 5:**
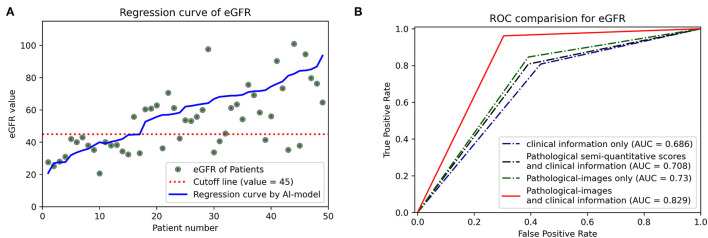
**(A)** Estimated glomerular filtration rate regression curves. The predictive values of 49 patients are arranged in ascending order, and the corresponding actual values (green dots) and their regression curves (blue lines) are indicated. Cutoff lines (red dashed lines) are also added to facilitate classification. **(B)** Receiver operating characteristic curves (ROCs) and areas under the ROC curve (AUCs) were used to show the binary classification results of the different models to predict whether eGFR is higher than 45 ml/min/1.73 m^2^.

Regression methods were employed to predict the stable eGFR, and the concordance between the predicted value and true label and the predictive accuracy are listed in Table 2. The EfficientNet-B5+CIE model showed the best predictive accordance and accuracy; it had the lowest MAE (12.52) and RMSE (16.67) and the highest R2 (0.38) and EVS (0.47). The regression curves for stable eGFR are displayed in Figure 5A. When classifying stable eGFR into dichotomies by the cutoff value of 45 ml/min/1.73 m^2^, the EfficientNet-B5+CIE model significantly improved the predictive performance from an AUC of 0.69 by the CIE model, 0.71 by the CIE+PRS model, and 0.73 by the EfficientNet-B5 model to an AUC of 0.83 by the EfficientNet-B5+CIE model (as shown in Figure 5B, Table 2). The EfficientNet-B5+CIE model showed good predictive ability, with a sensitivity of 0.96, a specificity of 0.70, a positive predictive value of 0.78, and a negative predictive value of 0.94.

**Table 2:** Prediction of postoperative stable graft function results.

		**Regression index**				**Classification index**						
**Target**	**Models**	**MAE**	**RMSE**	**R^**2**^**	**EVS**	**AUC**	**ACC**	**PPV**	**NPV**	**Sens**	**Spec**	**F1**
eGFR	EfficientNet-B5 + CIE	12.52	16.67	0.38	0.47	0.83	0.86	0.78	0.94	0.96	0.70	0.83
	EfficientNet-B5 (image)	14.68	19.32	0.31	0.41	0.73	0.74	0.71	0.78	0.85	0.61	0.73
	CIE+PRS	14.97	20.55	0.27	0.36	0.71	0.71	0.69	0.74	0.82	0.59	0.71
	CIE	15.07	21.37	0.25	0.35	0.69	0.70	0.68	0.72	0.81	0.57	0.69
	Inception-V3 (image)	15.92	22.58	0.21	0.29	0.71	0.72	0.71	0.83	0.85	0.62	0.71
	VGG19 (image)	18.42	28.18	0.12	0.16	0.64	0.65	0.61	0.73	0.79	0.53	0.65
RGF	EfficientNet-B5 + CIE					0.80	0.76	0.74	0.77	0.66	0.83	0.75
	EfficientNet-B5 (image)					0.71	0.74	0.77	0.72	0.61	0.81	0.70
	CIE+PRS					0.70	0.68	0.58	0.74	0.56	0.76	0.68
	CIE					0.66	0.66	0.62	0.68	0.55	0.77	0.64
	Inception-V3 (image)					0.65	0.73	0.61	0.68	0.56	0.76	0.65
	VGG19 (image)					0.59	0.61	0.56	0.66	0.45	0.75	0.57

*CIE, clinical information extraction (clinical characteristics); PRS, pathological Remuzzi scores; MAE, mean absolute error; RMSE, root mean squared error; R^2^, R-squared; EVS, explained variance score; AUC, area under the ROC curve; ACC, accuracy; PPV, positive predictive value; NPV, negative predictive value; Sens, sensitivity; Spec, specificity; F1, F1 score*.

*Classification of estimated glomerular filtration rate (eGFR) was divided from a cutoff value of 45 ml/min/1.73 m^2^. eGFR ≥ 45 ml/min/1.73 m^2^ was set as the target. Clinical information extraction (CIE) included listed clinical features, including donor characteristics, match and preservation features, and recipient characteristics, which are given in Table 1*.

**Figure 6:**
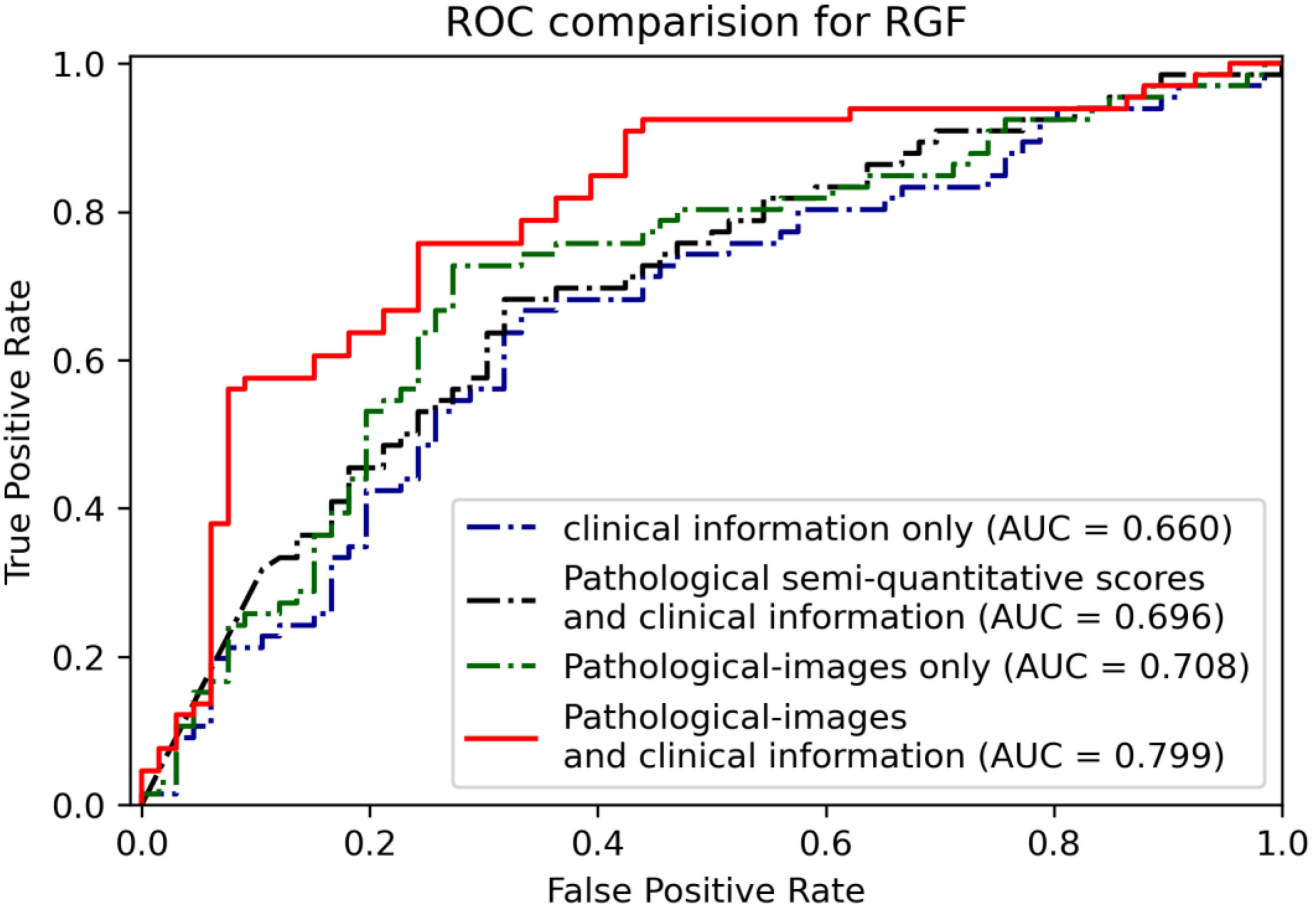
Receiver operating characteristic curves and AUCs show the binary classification results of different models for predicting RGF.

For RGF prediction, the EfficientNet-B5+CIE model had the best predictive performance. The pathological image plus clinical characteristics model significantly improved discrimination from an AUC of 0.66 by the CIE model and 0.71 by the EfficientNet-B5 model to an AUC of 0.80 by the EfficientNet-B5+CIE model (Figure 6, Table 2).

### Visualization

**Figure 7:**
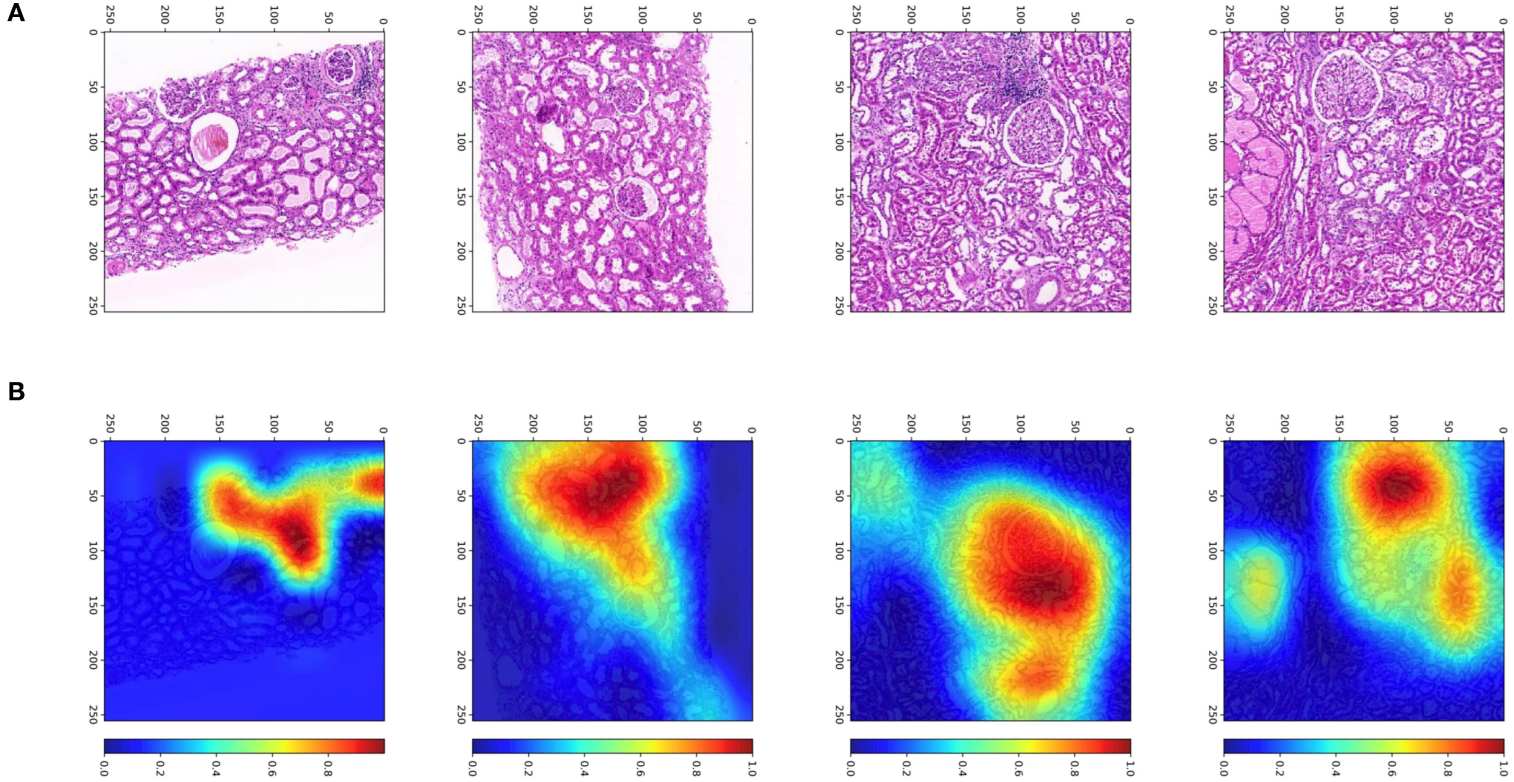
Patches and heatmap of RGF. **(A)** Patches from WSIs at × 200, resized to 256 pixels; **(B)** the corresponding gradient-weighted class activation mapping (Grad-CAM) heatmap, with red areas showing discriminative features for classification.

To determine from which areas the model extracts the most information, we used gradient-weighted class activation mappings (Grad-CAMs) to explain the results and display them visually. Grad-CAM can translate the output class into a final convolutional layer to produce a low-resolution map for a particular category (e.g., RGF) and highlight the discriminative image regions used by models to identify that category. With the help of Grad-CAM, we can judge whether the classification basis of the model is consistent with the clinical scenarios. Some examples of Grad-CAMs are illustrated in Figure 7. The models localized the areas of the tubules and interstitium near the glomeruli, which were discriminative features for classifying RGF from patches.

## Discussion

Artificial intelligence (AI) is being widely used in medical scenarios, and with tissue structure inception, there have been some advances in AI studies on renal microstructure histopathology in the last couple of years. For instance, the nephrology and computer science communities have been working on AI for kidney lesion segmentation (16–21). Jayapandian et al. (17) indicated that periodic acid-Schiff (PAS)-stained WSIs yielded the best concordance in deep learning segmentation. Hermsen et al. (18) presented a CNN for multiclass segmentation of PAS-stained kidney samples, with which the glomeruli, tubules, and interstitium were well-classified. Furthermore, Bouteldja et al. (19) used a deep learning algorithm for multiclass segmentation of PAS-stained kidney tissue WSIs; the trained CNN segmented six major kidney structures, including the glomerular tuft and glomerulus (such as, Bowman's capsule, tubules, arteries, arterial lumina, and veins), in various species and disease models. Additionally, Uchino et al. (20) developed AI models for seven major pathological lesions, global sclerosis, segmental sclerosis, endocapillary proliferation, mesangial matrix accumulation, mesangial cell proliferation, crescent, and basement membrane structural changes, with the AI model for global sclerosis showing excellent performance. Compared with experienced pathologists, Ligabue et al. (21) achieved a fast speed and comparable accuracy for kidney immunofluorescence reporting by a CNN. The aforementioned deep learning algorithms for glomerulus segmentation were based on PAS-stained or immunohistochemically stained slides. However, properly frozen biopsy slides are preferred in transplantation clinical practice. The CNN-based model developed by Marsh et al. (22) achieved good performance for glomerular sclerosis classification of frozen wedge biopsies. These results indicate the potential application of such models in donor kidney quality evaluation. Nevertheless, there is no study to date on machine learning for the relationship between donor biopsy images and transplant outcomes. Indeed, our study is the first to explore the power of AI on biopsy images and graft prognosis.

For this study, we used donor kidney biopsy WSIs as a feature in addition to clinical characteristics for graft function prediction. Compared with the net clinical characteristics model, the hybrid model displayed the lowest RMSE and MAE. When dividing eGFR by a cutoff of 45 ml/min/1.73 m^2^, the results showed distinct improvement in the prediction performance of the deep learning algorithms in addition to the clinical characteristics model for independent internal test data. Additionally, the addition of biopsy WSIs improved the prediction accuracy of RGF, and the AUC increased from 0.66 to 0.80. Overall, donor kidney biopsy WSIs are a useful predictor for graft function recovery. As graft and recipient long-term survival is impacted by posttransplant monitoring and treatment, graft function, complications, dnDSA, chronic rejection, it is difficult to predict long-term survival based on pretransplant donor and recipient parameters. Montero et al. (6) summarized previous prediction models for graft survival, reporting low prediction power in models based on pretransplant features. In general, pretransplant donor scores, such as the deceased donor score (DDS), donor risk score (DRS), SCD/ECD, and KDRI/KDPI, have limited predictive performance for graft survival. The discriminative ability (C-index) is approximately 0.6 (23, 24). However, adding posttransplant factors, such as eGFR, proteinuria, acute rejection, and allograft histological parameters, to prediction models significantly increases prediction accuracy (6), which indicates the importance of posttransplant management. Although posttransplant-based models have achieved excellent predictive performance, they are not suitable for pretransplant assessment and decision making. Among posttransplant factors, eGFR plays a critical role in graft survival and recipient quality of life. Functional or well-regenerated kidneys free recipients from dialysis, and a well-regernerated kidney congenitally determines the subsequent incidence of complications as well as the life span of the graft. Many studies have proven that well-regenerated renal function decreases the risk of long-term graft loss (2, 3, 15). Thus, renal function is deemed as a surrogate endpoint. In this study, we treated RGF and graft function as outcomes of early graft recovery. However, the time point that is suitable for eGFR assessment varies in previous studies (2, 3, 15). During the first 3~6 months, eGFR is not stable because of the need for antibiotics for infection prophylaxis, high-dose immunosuppression agents, and intensive rejection or infection occurrence. After 6 months, these events decrease, and eGFR gradually stabilizes. Most studies have used 1-year renal function as an indicator (2, 15) and even eGFR at 3 months (3). In this study, we employed a median value of a best period from each monitored eGFR during 3~12 months instead of eGFR at a certain time point. This was independently evaluated, and any obtrusive values with definite causes, such as rejection or infection were excluded.

Among previous DGF prediction models, Irish's DGF nomogram is widely accepted, but the dimensions of the model are complex; its predictive performance is moderate, with a C-index of 0.704 (25). Using bivariate cartography of DGF as an early graft function indicator is arbitrary. In addition, the decreased velocity of serum creatinine plays a critical role in non-DGF recipients. Non-DGF can be divided into SGF and IGF, and among numerous definitions for SGF and IGF, that using serum creatinine at postoperative day 7 (POD7) has the strongest correlation with 12-month graft eGFR (13). Thus, we adopted this definition in this study. Because the incidence of DGF was low and the sample size was small, machine learning with such an imbalanced dataset was hindered. Therefore, we merged the DGF and SGF groups into an RGF group as a surrogate endpoint, as based on a previous study (26). Our results showed a significant improvement in the predictive performance of RGF using the independent internal validation set. This result should be interpreted with caution because our sample size was relatively small, and we did not apply external validation. A multicenter cohort with a large sample size is needed for confirmation. Additionally, we performed heatmap analysis called gradient-weighted class activation mappings (Grad-CAMs) to explore on which feature the machine learning is focused. The results showed that Grad-CAMs focused on the tubules and interstitium near the glomeruli, which were discriminative features for RGF.

For graft function prediction, there is currently no stable predictive algorithm for graft eGFR after transplantation in deceased-donor recipients. The Nyberg score (DDS) is a 0~39 scoring system based on feature correlation with 6-month serum creatinine clearance (27). DDS includes donor and HLA matching characteristics, such as donor age, terminal creatinine clearance (by the Cockcroft-Gault equation from age, sex, weight, and terminal serum creatinine), donor hypertension, cause of death, and HLA mismatch for donor quality assessment. Rhu et al. (28) developed a linear model for the prediction of recipient creatinine levels in living-donor transplantation. This linear model incorporates characteristics, such as donor age, donor height, donor serum creatinine, graft weight, recipient sex, recipient height, and recipient weight, showing an R^2^ of 0.708, an RMSE of 0.161, and an intraclass correlation coefficient (ICC) of 0.83. In addition, the model achieved good prediction performance in the external validation cohort. In another study, Lasserre et al. (7) utilized machine learning approaches to predict transplant graft function outcomes (eGFR after 1 year) *via* classic clinical characteristics. The Gaussian support vector machine with recursive feature elimination is best for predictions, with a correlation coefficient of 0.48. When defining transplantation failure with eGFR <45 ml/min, the AUC was 0.726. Our model using simple clinical features had a similar AUC of 0.69. Sexton and colleagues tested the discrimination of KDRI/KDPI for eGFR in the Ireland population, and according to the results, KDRI/KDPI was significantly associated with eGFR over 5 years but only accounted for 21% of eGFR variability over time (24). Overall, the predictive performance of using net clinical features is relatively poor. In this study, a distinct improvement in eGFR classification performance was obtained by using deep learning algorithms for pretransplant biopsy images combined with the clinical characteristics model, with the AUC increasing from 0.69 to 0.83. When using biopsy images alone, the AUC of deep learning reached 0.73 and surpassed that of the clinical feature model. Although exact prediction (perfect fitting) of graft eGFR is difficult to achieve for deceased-donor recipients, good classification of eGFR can be accurate.

Some limitations exist in this study. First, we used FFPE H&E-stained WSIs, which differ from frozen H&E-stained biopsy in terms of slide quality and staining effect. Traditional FFPE H&E staining preserves morphology with few artifacts but also requires a long processing time. In our center, at least 12 h were needed to complete the whole FFPE H&E-stained slide preparation, and it is not suitable for the emergent evaluation of donor kidneys due to time constraints. Fast paraffin blocks can be prepared within several hours and is an alternative. Second, this was a retrospective study, and selection bias was inevitable. Pretransplantation biopsy usually occurs in expanded donation or procedural biopsy. Third, ground-truth label imbalance had a negative impact on the predictive model. The number of recipients with eGFR <45 ml/min/1.73 m^2^ was much lower than the number for the eGFR≥ 45 ml/min/1.73 m^2^ group. Thus, the model favored the prediction of eGFR ≥ 45 ml/min/1.73 m^2^. Additionally, we labeled only the glomeruli and did not clarify the microstructures of the WSIs in terms of the glomeruli, tubules, and interstitium, which would probably provide more detailed information and improve prediction accuracy. Due to the short follow-up times, we did not perform a long-term survival analysis.

Despite the aforementioned limitations, our results preliminarily show that the addition of deep learning for FFPE H&E-stained WSIs improves the accuracy of graft function prediction in deceased-donor kidney transplant recipients. However, a multicenter prospective study based on frozen biopsy or fast paraffin H&E-stained WSIs incorporating detailed microstructure recognition is needed for validation.

## Data Availability Statement

The original contributions presented in the study are included in the article/supplementary material, further inquiries can be directed to the corresponding author/s.

## Ethics Statement

The studies involving human participants were reviewed and approved by Institute Review Board of the Third Affiliated Hospital of Sun Yat-sen University. The Ethics Committee waived the requirement of written informed consent for participation.

## Author Contributions

NN and YR conceived of and designed this study. YL, JL, ZT, JZ, ZD, WD, and BM collected the data and biopsy slides. LH, YR, and YL analyzed the data. YL and XH wrote the manuscript. BM and NN supervised and revised the manuscript. All authors contributed to the article and approved the submitted version.

## Funding

This work was supported by the National Natural Science Foundation of China (81970652 and 81702409), the Guangdong Basic and Applied Basic Research Foundation (2019A1515011219), the Bioengineering Research Center Training Project of the Third Affiliated Hospital of Sun Yat-sen University (SW201904), the Third Affiliated Hospital of Sun Yat-sen University Clinical Research Program (YHJH201906), the Science and Technology Planning Project of Guangzhou (201803010016), and the Guangdong Provincial Key Laboratory of Digestive Cancer Research (No. 2021B1212040006).

## Conflict of Interest

The authors declare that the research was conducted in the absence of any commercial or financial relationships that could be construed as a potential conflict of interest.

## Publisher's Note

All claims expressed in this article are solely those of the authors and do not necessarily represent those of their affiliated organizations, or those of the publisher, the editors and the reviewers. Any product that may be evaluated in this article, or claim that may be made by its manufacturer, is not guaranteed or endorsed by the publisher.

## Abbreviations

WSIs: Whole-slide images
CIE: clinical information extraction
H&E: hematoxylin-eosin
PAS: periodic acid-Schiff
eGFR: estimated glomerular filtration rate
CNN: convolutional neural network
MAE: mean absolute error
RMSE: root mean square error
ROC: receiver operating characteristic
DGF: delayed graft function
SGF: slow graft function
AI: artificial intelligence
COTRS: China Organ Transplant Response System
ROIs: regions of interest
ECD: expanded criteria donor
KDRI: Kidney Donor Risk Index.
